# Multi-site harmonization of 7 tesla MRI neuroimaging protocols

**DOI:** 10.1016/j.neuroimage.2019.116335

**Published:** 2019-11-08

**Authors:** William T. Clarke, Olivier Mougin, Ian D. Driver, Catarina Rua, Andrew T. Morgan, Michael Asghar, Stuart Clare, Susan Francis, Richard G. Wise, Christopher T. Rodgers, Adrian Carpenter, Keith Muir, Richard Bowtell

**Affiliations:** a Wellcome Centre for Integrative Neuroimaging, FMRIB, Nuffield Department of Clinical Neurosciences, University of Oxford, Oxford, United Kingdom; b Sir Peter Mansfield Imaging Centre, School of Physics and Astronomy, University of Nottingham, Nottingham, United Kingdom; c Cardiff University Brain Research Imaging Centre, School of Psychology, Cardiff University, Cardiff, United Kingdom; d Wolfson Brain Imaging Centre, Department of Clinical Neurosciences, University of Cambridge, Cambridge, United Kingdom; e Imaging Centre of Excellence, University of Glasgow, Glasgow, United Kingdom

**Keywords:** 7 tesla, MRI, Harmonization, Anatomical, Functional, Scanner calibration

## Abstract

Increasing numbers of 7 T (7 T) magnetic resonance imaging (MRI) scanners are in research and clinical use. 7 T MRI can increase the scanning speed, spatial resolution and contrast-to-noise-ratio of many neuroimaging protocols, but technical challenges in implementation have been addressed in a variety of ways across sites. In order to facilitate multi-centre studies and ensure consistency of findings across sites, it is desirable that 7 T MRI sites implement common high-quality neuroimaging protocols that can accommodate different scanner models and software versions.

With the installation of several new 7 T MRI scanners in the United Kingdom, the UK7T Network was established with an aim to create a set of harmonized structural and functional neuroimaging sequences and protocols. The Network currently includes five sites, which use three different scanner platforms, provided by two different vendors.

Here we describe the harmonization of functional and anatomical imaging protocols across the three different scanner models, detailing the necessary changes to pulse sequences and reconstruction methods. The harmonized sequences are fully described, along with implementation details. Example datasets acquired from the same subject on all Network scanners are made available. Based on these data, an evaluation of the harmonization is provided. In addition, the implementation and validation of a common system calibration process is described.

## 1 Introduction

The number of magnetic resonance imaging (MRI) scanners operating at 7 T world-wide now exceeds 70 (Huber, 2018). Neuroimaging researchers’ access to 7 T scanners is therefore increasingly common. At the same time 7 T MRI is becoming available for clinical use, with commercially available models achieving regulatory certification for neuroradiology in both America and Europe (US Food & Drug Administration, 2017).

7 T MRI has the potential to bring increased scanning speed, spatial resolution and contrast-to-noise-ratio to a wide range of neuroimaging protocols (Trattnig et al., 2016). Nevertheless, significant technical challenges such as increased inhomogeneity in the B_0_ and radio frequency (RF) fields and increased specific absorption ratio (SAR) burden mean that protocols used at lower field-strengths have to be adapted for implementation at 7 T (Kraff and Quick, 2017; Garwood et al., 2001). The process of modifying and optimising an entire imaging protocol for 7 T requires expert physics, radiography and neuroradiology input and is therefore often site- and hardware-specific.

With the increased availability of 7 T scanners, especially within clinical environments, there is a need to ensure that, despite the varying models, suppliers, hardware and software, sites have the capability to:

a) implement full protocols required for standard neuroimaging,
b) collect reproducible data, and
c) compare data meaningfully between sites.

In the United Kingdom (UK) the number of 7 T MRI scanners has increased from two to five in the last two years, with a further two scanners to be installed in the near future. The five operational scanners include three different models of scanner, provided by two different vendors. The UK7T Network was established with a specific aim to create a set of harmonized structural and functional neuroimaging sequences and protocols that would provide all of the UK's 7 T sites with the above-mentioned capabilities.

Here, we describe the process by which the sequences and reconstruction processes were harmonized on three different scanner platforms. The harmonized sequences are described, along with implementation details. Example datasets from a single subject that were acquired on the three different scanner models are also made available. Based on these data, both a qualitative, and a simple quantitative, evaluation of harmonization are provided. In addition, the implementation and validation of a common system calibration process is described.

## 2 Methods

### 2.1 Scanners & equipment

The MRI scanners used in this work are located at five UK university research facilities. They comprise three different models of 7 T scanner. The location and technical specification of each scanner is given in Table 1.

Each scanner was equipped with a Nova Medical (Wilmington MA, USA) single-channel transmit, 32-channel receive (1Tx32Rx) head coil. All protocols were optimised for use with this coil.

### 2.2 Choice of sequences

Harmonization focussed on well-established structural and functional neuroimaging sequences. Sequences for T_1_, T_2_ and T_2_* weighted imaging were included for structural imaging. Functional imaging utilised gradient echo EPI sequences for measurement of BOLD contrast. Scanner characterisation and calibration sequences (B_0_ and flip-angle mapping) were also included and harmonized. Precise sequence choice was guided by availability in the scanner sequence library, established use cases, and ability to make required modifications. Sequences that exploit parallel transmit (pTx) capability were not considered at this stage since pTx hardware and sequences were not available on all scanners.

T_1_ weighted imaging was based upon using MPRAGE-type sequences (Marques et al., 2010; Mugler and Brookeman, 1990). Both MPRAGE and MP2RAGE sequences were included and were used for tissue segmentation. Both single and multi-echo gradient echo (GRE) sequences were included for T_2_* weighted imaging. T_2_ weighted imaging was achieved by using a variable flip-angle turbo-spin-echo sequence. T_1_ and T_2_* weighted imaging sequences were harmonized for whole brain imaging. Due to the significant ${\text{B}}_{1}^{+}$ inhomogeneity effects that are present in images acquired using spin echo sequences when single-channel transmit coils are used at 7 T, T_2_ weighted imaging was harmonized for a restricted brain region only.

Both single and multi-band GRE-EPI imaging sequences were included. The vendors' own single band EPI sequences were utilised. For multi-band EPI imaging, the CMRR (University of Minnesota, Minnesota, USA) implementation (Moeller et al., 2010) was used on Siemens platforms due to cross-model compatibility. The site's own implementation was used on the single Philips scanner.

Spin-echo EPI variants of each sequence were included for EPI distortion correction (9). The choice of distortion correction method was made as a result of previous work (Driver et al., 2018), which was corroborated by further measurements made in this study (see Section 2.7).

EPI sequences were separately harmonized for whole brain functional connectivity measurements and brain activation during motor and visual fMRI experiments. In both cases an isotropic resolution of 1.5 mm was chosen, with a repetition time of ≤2 s.

A dual-echo GRE sequence was used for B_0_ mapping. 3D DREAM flip-angle mapping was used due to its short whole-head acquisition time and its availability on all systems (Nehrke and Bornert, 2012; Ehses et al., 2019).

### 2.3 Harmonization - sequences

Three aspects of the pulse sequences were harmonized: the sequence program, the sequence parameters and the radio-frequency pulses used for adiabatic inversion. Where possible, the same sequence program was used. This was always possible on scanners of the same model, and typically possible across scanners of the same vendor. Between vendors, harmonization of the same category of sequences (e.g. gradient recalled echo [Siemens] and fast field echo [Philips]) was achieved in the following way.

Core sequence parameters were set to be equal across all scanner models. This includes, but is not limited to: spatial resolution, field of view, slice/slab orientation, sequence timings (repetition time – TR, echo time – TE, inversion time – TI and echo-spacing), bandwidth, flip-angle, fat-saturation scheme and flow compensation. If equalisation was not possible, e.g. due to an imposed step size, the values were set to be as close as possible. Values were set to match those used in existing optimised protocols at the network sites, e.g. Mougin et al. (2016).

Finally, key radiofrequency pulses were matched exactly. In the T_1_ weighted MP2RAGE sequences the same optimised inversion pulse was incorporated into the sequence on all scanner models (Hurley et al., 2010). In the multiband GE-EPI sequences, pulse durations were matched.

Several aspects of pulse sequences were not harmonized. No attempt was made to harmonize the in-plane parallel acceleration methods, therefore on the Philips scanner SENSE acceleration was used and GRAPPA acceleration was used on the Siemens scanners (Griswold et al., 2002; Pruessmann et al., 1999), but the in-plane acceleration factor was matched across all scanners. Precise gradient waveforms were also not also matched. Scanner calibration steps were not modified, although some were superseded by manual intervention (see Section 2.5).

The EPI sequences for task-fMRI were optimised for specific fMRI protocols: a block design motor-visual protocol and a four-digit phase-encoded motor protocol. Details are given in Appendix A and computer code for visual cuing of the protocols is available with the harmonized sequences at Clarke (2018).

### 2.4 Harmonization - reconstruction

The vendor's own, or sequence specific program was used for image reconstruction. Optional “black box” reconstruction steps (filters and normalisation steps) were disabled, where possible.

To enable meaningful phase imaging on Siemens systems, the reconstruction step in which signals from different receiver coils are combined was reimplemented. To match the SENSE-based coil combination approach used on the Philips system as closely as possible, a Roemer combination (SENSE R = 1) algorithm was implemented on the Siemens systems as described in Roemer et al. (1990) and in Appendix B. In short, low resolution GRE images were acquired using the coil's array and volume elements and used to calculate coil sensitivity profiles for each subject. The coil sensitivity image was processed and fitted with polynomials in the spatial domain as described in Pruessmann et al. (1999) and stored in memory for use in reconstructing images from subsequent acquisitions on the same subject. A uniform sensitivity combination was used to replace the vendor's own sum of squares or “Adaptive Combine” combination in sequences requiring a phase image output and in the MPRAGE scan (Walsh et al., 2000).

### 2.5 Assessment of harmonization

The level of harmonization of the sequences was tested by scanning the same subject (male, 28 years old, 82 kg) at all five sites. In addition, the subject was scanned an additional four times at one site (site #1) to assess inter-session reproducibility. Different sequence contrasts were assessed using specific metrics, as described below.

T1w data were segmented after carrying out offline PSIR (phase-sensitive inversion recovery) reconstruction on all MP2RAGE data, including a simple method to denoise non-tissue pixels (Mougin et al., 2016; O'Brien et al., 2014). Additionally, the MP2RAGE data were segmented using the Freesurfer longitudinal stream after inhomogeneity-correction using SPM (Ashburner and Friston 2005; Reuter et al., 2012). The mean values of cortical thickness in the right and left post-central gyri were compared across sessions and sites using a Wilcoxon signed-rank test, while the variances of these measures were compared using a Brown-Forsythe test. Coefficients of variation (CoV) were computed for each region output from the DKT Atlas (Klein and Tourville, 2012). Dice Similarity Coefficients (DSC) were also computed on the cortical ribbon segmentation, comparing the segmentations from the nine scans to the segmentation of the average subject space.

Multi-echo GRE data were used to calculate mono-exponential R2* relaxation rates using the Auto-Regression on Linear Operations (ARLO) method (Pei et al., 2015). Single-echo GRE images were phase unwrapped and underwent background field removal (using a two-step LBV [Laplacian Boundary Value] and vSMV [variable spherical mean value] approach) before being used to generate quantitative susceptibility maps (QSM) using the QSMBox v2.0 (MSDI method, self-optimised scale, λ = 10^2.7^) (Acosta-Cabronero et al., 2018; Li et al., 2011; Zhou et al., 2014; Abdul-Rahman et al., 2005). In each case, quantitative values were extracted from five subcortical regions of interest (ROI), namely the: substantia nigra, red nucleus, globus pallidus, putamen and caudate (Lin et al., 2015; Santin et al., 2017). Standard deviation across scans of the average value within each ROI was used to quantify inter-site and inter-session variability.

Percentage BOLD activation and extent during block motor-visual stimulation was used to assess harmonization of the multi-band EPI sequence used for the task-based fMRI. The data were motion corrected (FSL MCFLIRT(31)) and distortion corrected (FSL topup (9)). Task activation was then assessed using FSL FEAT (Woolrich et al., 2001)(FDR-corrected p_FDR_<0.01). Analysis of activation was spatially restricted by task to the occipital lobe for visual stimulus, the right pre- and post-central gyri for left-hand movement and the left pre- and post-central gyri for right-hand movement (Harvard-Oxford atlas (Desikan et al., 2006)). The CoV in BOLD percentage change (%BOLD) and extent of activation was calculated across the five sessions between sites for inter-site variation, or the five sessions at the same site for intra-site variation. To compare %BOLD across sites, a common ROI based on an intersection mask formed from the data acquired at all five sites was generated.

The motortopy task entailed a visually paced sequential button press of 8 s blocks of digit movement, cycling across digit blocks. Data sets were acquired in both forward (from index finger to little finger) and reverse (from little finger to index finger) ordering (see Appendix A). A Fourier-based travelling wave analysis was performed to calculate voxel-wise phase and coherence of the BOLD response to the motortopy task using mrTools, following the methods detailed in Sanchez-Panchuelo et al. (Sanchez-Panchuelo et al., 2010; Gardner et al., 2018). Voxel-wise phase values from the travelling wave motortopy task were compared across sessions both in a pairwise manner, by polar histogram, and by calculating circular variance across sessions, using the Matlab Circular Statistics Toolbox (Berens, 2009).

Four-hundred volumes of resting-state fMRI (rs-fMRI) data were collected during each functional scanning session (see Table 3). Participants were asked to focus on a fixation cross (approximately 2° visual angle) appearing on a black screen, with the scanning room lights off. rs-fMRI data were quantitatively assessed using temporal SNR (tSNR) measurements. Data were motion corrected with FSL MCFLIRT (Jenkinson et al., 2002) and distortion corrected with FSL topup (Andersson et al., 2003). Temporal mean, standard deviation and tSNR images were computed. A grey matter mask was defined for the cerebrum, which was segmented into frontal, parietal, occipital and temporal lobes. Additional ROIs were defined for the cerebellum and subcortex (restricted to brain regions covered by all site protocols). The mean and standard deviation of tSNR values were calculated for each session within ROIs. To assess inter-session and inter-site tSNR differences, tSNR distributions were estimated using a bootstrap method. The mean tSNR value for each ROI in each scan were resampled (sub-sampled) 100 times. From the resultant discrete distribution of ROI means, the continuous distributions were determined using kernel density estimation with a Gaussian kernel (of bandwidth = 4). Inter-session and inter-site values were compared statistically with Wilcoxon rank-sum tests.

### 2.6 System calibration

To obtain the same contrast and signal-to-noise ratio (SNR), the transmit RF calibration of each scanner model had to be harmonized to achieve the same flip-angle distribution on a given subject. After initial calibration using vendor-specific methods, a 3D DREAM sequence was used to measure voxel-wise flip-angles in the subject's brain. Vendor-specific methods vary in measurement technique and degree of spatial localisation. Subsequently a whole brain mean flip-angle was estimated from a single axial slice and used to adjust the transmit calibration (Clarke et al., 2018). The adjustment protocol is included in Appendix C.

For most scans the vendor's own automatic B_0_-shimming process was found to be sufficient with little variation in results between scanner models. For sequences that are particularly prone to off-resonance artefacts (e.g. EPI), the vendor's automatic shimming was iterated twice. On the Siemens Magnetom Terra (Sites 4 & 5 in Table 1) there was an additional requirement for manual adjustment. At these sites manual “iterative shimming” of the linear-z shims was performed to minimise measured linewidth.

The effectiveness of the harmonized system calibration was assessed by analysing the results of whole brain dual-echo GRE B_0_ mapping and DREAM flip-angle mapping in three identical subjects on each scanner model.

### 2.7 EPI distortion correction

EPI distortion correction efficacy was assessed for three different methods at 7 T:

1. Correction using FSL's topup using reversed phase-encode-blip, spinecho EPI volumes (Andersson et al., 2003),
2. Correction using FSL's topup using reversed phase-encode-blip, GRE EPI volumes,
3. EPI unwarping using conventional dual-echo GRE derived B_0_ field maps (FSL FUGUE) (Jenkinson, 2004).

Each method was used to correct a pair of reversed phase-encode-blip GRE-EPI volumes acquired using the parameters of the “Resting state fMRI” protocol in Table 3. An assessment of distortion correction efficacy was made by calculating the root-mean-square difference (RMSD) of the corrected “blip-up” & “blip-down” volumes. The images were masked using FSL BET before the calculation (Smith, 2002). Effective distortion correction should minimise differences between two otherwise identical EPI volumes acquired sequentially. This analysis was performed using data acquired on the same subject as in Section 2.5 across all five scanners, including repeats at Site #1. B_0_ field-maps were acquired as described for the “structural” protocol in Table 2.

## 3 Results

### 3.1 Harmonized protocols & data release

Tables 2 and 3 list the harmonized sequences and summarises the relevant parameters of each sequence. The sequence parameters can be found in full at Clarke (2018). This information is accompanied by details of the necessary implementation steps, sequence versions and a description of any modifications made to the normally available (e.g. vendor's default) sequence. Source code of modified sequences and reconstruction pipelines is available by agreement.

The DICOM (Association NEM, 1205) and NIfTI format data from the single subject assessment of the harmonized scans are made available at Clarke (2018).

### 3.2 Qualitative assessment of harmonization

**Fig. 1:**
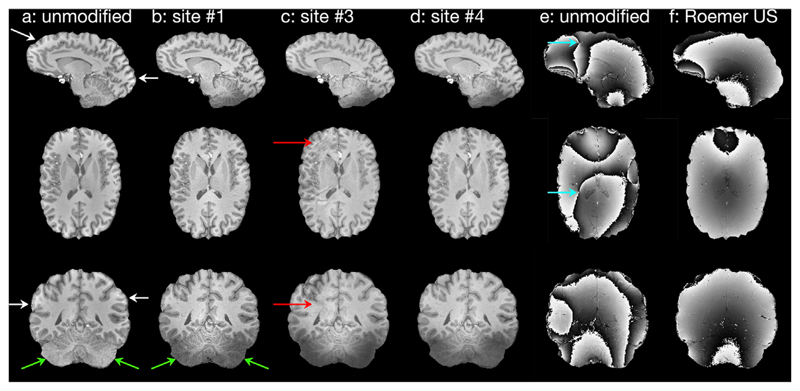
Harmonization of the MPRAGE sequence. **a** Original vendor implementation at Site 1 shows loss of contrast due to receive coil sensitivity profile (white arrows). **b-d** UK7T Network harmonized sequence at Sites 1,3&4 show improved and consistent contrast in the cerebellum, despite low ${\text{B}}_{1}^{-}$ sensitivity (green arrows) and homogenous receive sensitivity in the cortex. Some scanner specific artefacts are still visible e.g. SENSE ghost (red arrows). **e** Original vendor implementation at Site 1 shows singularities in the phase data (blue arrows). **f** The harmonized uniform sensitivity Roemer combination produced phase images free of open-ended fringe lines.

During the harmonization process the level of harmonization was assessed qualitatively. For example, the effect of harmonization on the MPRAGE sequence can be seen in Fig. 1. In this sequence, in addition to the sequence parameters the inversion pulse and coil combination was also harmonized. Good grey-white matter contrast can be observed throughout the brain of the single test subject on all scanners, including in the cerebellum. In comparison, default sequences showed some loss of contrast in inferior regions of the brain (Fig. 1a). The implemented uniform sensitivity Roemer coil combination produced phase images free of open-ended fringe lines, as seen in the Siemens’ default combination (Fig. 1e) and reduced receive coil sensitivity induced contrast differences (Fig. 1a).

**Fig. 2:**
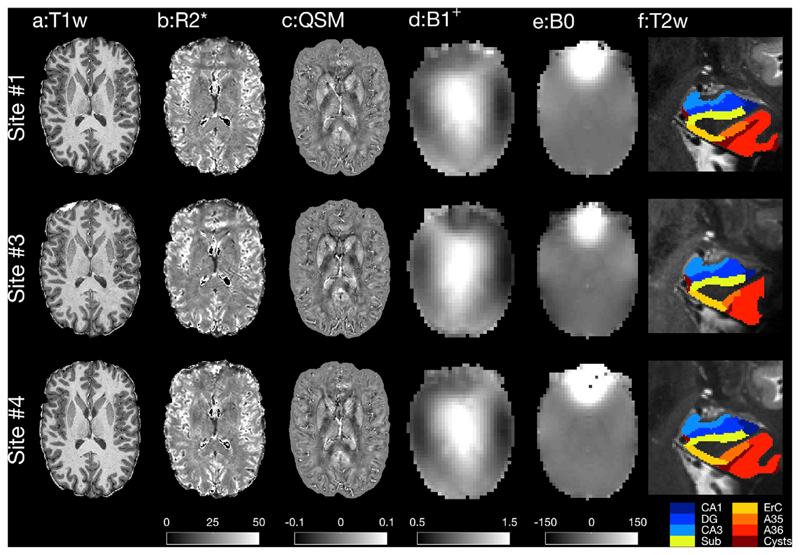
Example data from the UK7T Network harmonized structural sequences. Acquired on a single subject and on each model of scanner in the Network (Sites 1,3&4). **a** PSIR reconstruction of MP2RAGE. **b** R_2_* maps derived from the multi-echo GRE sequence. Units: Hz. **c** Quantitative susceptibility maps derived from single-echo GRE sequence. Units: ppm. **d** Calibrated DREAM flip-angle maps. Units: flip-angle normalised to target. **e** Dual-echo B_0_ maps. Units: Hz. **f** Results of automated hippocampus segmentation (by ASHS (Yushkevich et al., 2015; Berron et al., 2017)) using the TSE sequence.

**Fig. 3:**
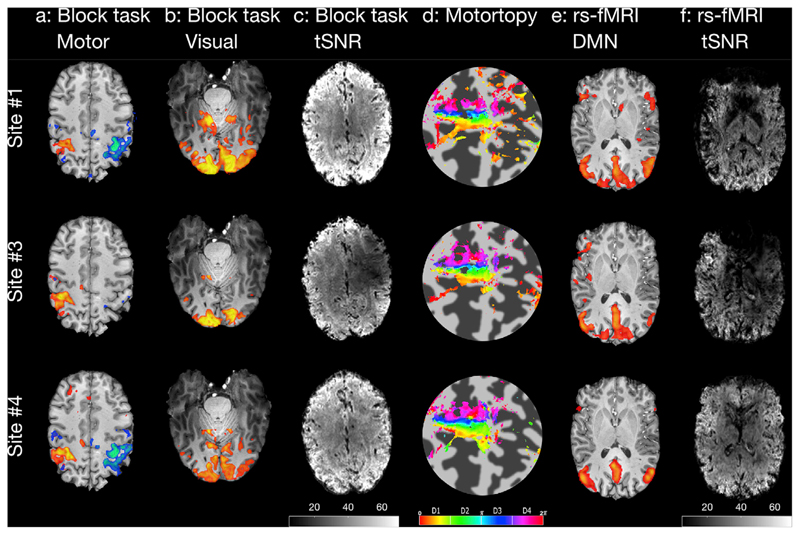
Example data from the UK7T Network harmonized functional sequences. Acquired on a single subject and on each model of scanner in the Network (Sites 1,3&4). **a** Threshold z-statistic maps (P < 2.3 × 10^−4^) of an alternate hand finger apposition task overlaid on the subject average MPRAGE. **b** Threshold z-statistic maps (P < 2.3 × 10^−4^) of a checkerboard visual presentation stimulus acquired simultaneously with **a**. **c** tSNR maps of the multi-band GRE-EPI scans used to calculate **a** & **b** (scale 5–70). **d** Phase plots of the phased motortopy task overlaid on a flat-map of the central sulcus. **e** Per-subject Default Mode network derived from dual regression of the single example subject's scans at all sites (Nickerson et al., 2017). **f** Axial tSNR maps of the multi-band GRE-EPI scans used to calculate e (scale 5–70).

Fig. 2 shows example structural images acquired on the single test subject using the harmonized protocols on three of the scanners in the study, comprising one of each of the scanner models. Contrasts provided by these structural sequences are seen to be similar with low artefact levels. The largest differences are observed in the system calibration scans, but it should be noted that these images were obtained before the manual calibration steps were performed. Preliminary analysis results from functional sequences are shown in Fig. 3. Both task and resting-state fMRI results show qualitatively similar activation profiles. However, temporal SNR (tSNR) maps show data quality differences remain.

### 3.3 Quantitative assessment of harmonization

**Fig. 4:**
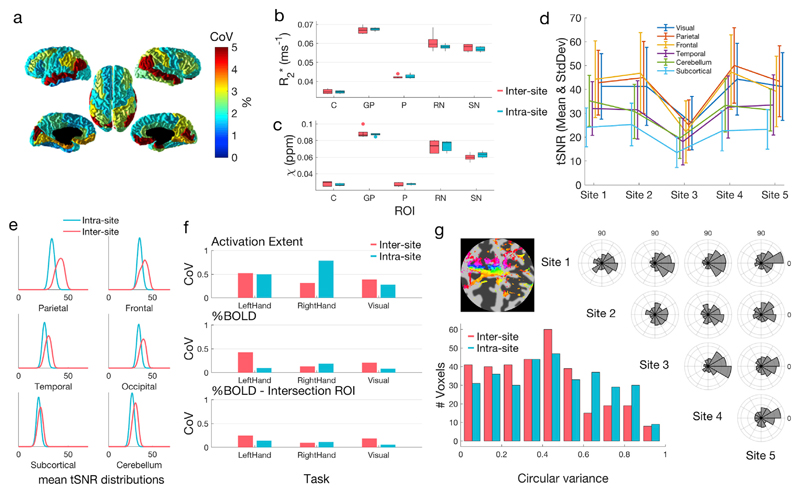
Quantitative evaluation of harmonization success for six contrasts. **a** Coefficient of variation in cortical volume as estimated from MP2RAGE images in Freesurfer for cortical ROIs. **b&c** Box plots of susceptibility, R_2_* (**b**) and χ (**c**) and values within five regions of interest across sites 1–5 (inter-site) and across five repetitions at site 1 (intra-site). ROIs: C = caudate, GP = globus pallidus, P = putamen, RN = red nucleus and SN = substantia nigra. **d** tSNR values for rs-fMRI data across sites 1–5 (inter-site). **e** Comparisons of inter-site and inter-session tSNR distributions in 6 brain regions. **f** Coefficient of variation of spatial activation extent and % BOLD change in ROIs for all stimulus tasks in the block task design. **g** Phase difference polar histograms between measurements made at each site, calculated across all voxels in a standard space ROI. The intra- and inter-site circular variance distributions are shown in the histogram. Low circular variance indicates high similarity between sessions.

No significant difference (p > 0.11, Wilcoxon signed-rank & Brown-Forsythe test) was detected in the means or variance of the left and right post-central cortical thickness (derived from T1w images) when measured within site or across sites. Coefficients of Variation (CoV) were computed for the cortical thickness measured by Freesurfer for each region from the DKT Atlas (Fig. 4a), and all CoV values were below 5%.

Cortical ribbon segmentation similarity (measured using the DICE Similarity Coefficient) was not significantly different for within site repeats (p = 0.43, Wilcoxon signed-rank). However, a significant difference was detected in the variance (p = 0.03, Brown-Forsythe test), with the across site scans showing a 40-times greater variance than the within-site repeats. After acquisition and processing of the data it was found that gradient non-linearity geometry correction was applied differently at Site 3 compared to all other sites on T1w data. Retrospective correction of this difference made the difference in variance non-significant in the above test.

Mono-exponential R2* values calculated from multi-echo GRE data in five regions of interest (Fig. 4b) showed no significant difference (Wilcoxon signed-rank test, p > 0.05) between values measured within site (five repeats at site 1) and across sites (1 scan at all five sites). On average the mean values in the ROIs across sites were 1% lower compared to the average within site value, though the standard deviation of the values measured across the sites was on average 101% larger than the within site standard deviation. The same analysis was made on the quantitative susceptibility values, χ, obtained from the single-echo GRE data (Fig. 4c). Once again, there was no significant difference, mean values were 1% larger, and standard deviations 86% larger.

We assessed the resting-state fMRI data by calculating tSNR values across six regions of the brain (Fig. 4d). We found differences in tSNR values between sites, but inter-site differences were not statistically different from inter-session differences in tSNR (Fig. 4e; Wilcoxon rank-sum test, p > 0.15 for all ROIs).

Coefficients of variation for inter-site and intra-site activation extent, %BOLD using a session-specific ROI and %BOLD using the intersection ROI are shown in Fig. 4f. Activation extent shows the highest CoV, on average 51% across all tasks, whilst %BOLD (intersection ROI) CoV is lower, on average 13%. The average inter-site CoV across all measures is 28%, the average intra-site CoV across all measures is 25%. There is no significant difference (Wilcoxon signed-rank test, p > 0.05) in the underlying intra- and inter-site distributions of all nine reported measures.

Motortopy phase difference histograms between sites show phase differences clustered around 0/360° (Fig. 4g). The intra- and inter-site circular variance distributions demonstrate that there is no more variance between inter-site measurements of digit motortopy maps than between repeated measurements at the same time (intra-site).

### 3.4 System calibration

**Fig. 5:**
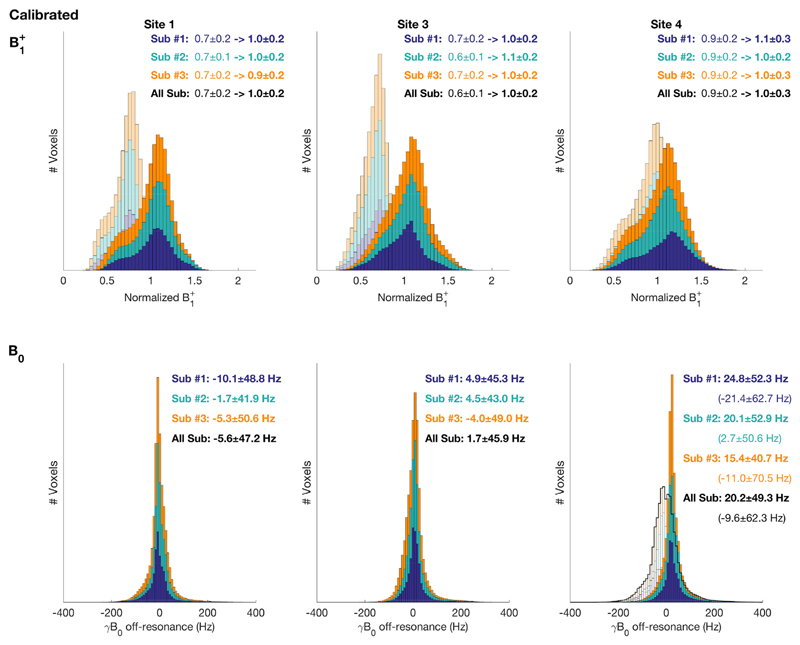
Whole-brain voxel-wise distributions of scanner calibration (${\text{B}}_{1}^{+}$ and B_0_) measured on three subjects. Top: The effect of manual calibration of transmitter gain. The faint histogram shows the results of the vendors' own calibration. The solid histogram shows the distribution after calibration using DREAM flip-angle maps. Bottom: B0 distributions. At site 4 & 5 (5 not shown) the distribution after the vendors automatic calibration is noticeably broader than the other scanner models (faint & black outline). A satisfactory shimming result is seen after manual shimming of the linear z gradient (solid histogram).

Fig. 5 shows the whole-brain distributions of normalised ${\text{B}}_{1}^{+}$ and B_0_ measured from three subjects on each model of scanner (Sites 1, 3 & 4). Prior to manual calibration of the ${\text{B}}_{1}^{+}$-field amplitude there was both a greater difference between models and a larger underestimation of the required calibration value than after calibration. Before calibration the range of mean values was 0.6–0.9 with an average of 0.73, after calibration all site means were 1.0.

After implementation of vendor-supplied whole-brain B_0_-shimming, equivalent performance was observed at Sites 1, 2 and 3. After default calibration at Sites 4 and 5 lower performance was observed, but if manual shimming is performed the standard deviation of the voxel-wise B_0_-field distribution decreases and equivalent performance with the other sites is achieved.

### 3.5 EPI distortion correction

Across all nine sessions the mean whole brain masked RMSD of Method 1 (SE-EPI) was lower (5 ± 3%), but not significantly (*t*-test, p = 0.32), than that of Method 3 (field-map). The mean RMSD of Method 1 was significantly lower (23 ± 7%, *t*-test, p = 0.03) than that of Method 2 (GRE-EPI).

Restricting the analysis to five transverse slices immediately superior of the ethmoid sinuses, Method 3 (field-map) resulted in significantly lower mean RMSD than Method 1 (SE-EPI)(31 ± 7%, *t*-test, p = 0.01) and Method 2 (GRE-EPI)(21 ± 9%, *t*-test, p = 0.03).

## 4 Discussion

### 4.1 Use of the protocols

These harmonized protocols have been developed to ensure that the UK7T Network's scanners are able to acquire comparable high-quality data. This is done with the aim of allowing future implementation of multi-centre patient studies and comparability of data across sites. The harmonized sequences will also form the basis of research protocols for other studies at the participating sites.

As the network covers many currently available models of human 7 T scanner, the protocols and example data are released for use at other 7 T-capable sites.

In addition, the protocols will be used in a future UK7T Network study, scanning dementia patients at each of the sites. The harmonized T_1_ weighted sequences are also being used to acquire T_1_ weighted scans from a large normative cohort, which will form a data-set for future release.

### 4.2 Validation of harmonized protocols

In this study we have carried out a limited validation of the harmonized protocols, looking at the results from a single subject. The cross-site CoV of cortical thickness derived from the T1w images is below 5% in all cortical regions. We also observe very small, non-significant inter-site differences in mean quantitative values in sub-cortical ROI analysis of susceptibility and R_2_* values derived from GRE data.

The analysis of the block-task stimulus, motortopy tasks and resting-state fMRI demonstrates that there is no increased variance in measurements taken across sites compared to repeated measurements at a single site. This indicates that the harmonization of the protocols is successful to the level of scan-to-scan variation on a single subject.

Quantitative analysis has highlighted areas requiring further harmonization effort or correction in data post-processing. Careful analysis of T1w and QSM data has highlighted differences in gradient non-linearity correction between vendors. Differences in tSNR performance of multiband EPI sequences highlight the difficulty of harmonising such sequences given their complex design, parameterisation and reconstruction – further hampered by issues of current vendor proprietorial interests.

The full efficacy of this harmonization effort is being assessed in an ongoing multi-centre “travelling-heads” study. Data acquisition, involving scanning of the same ten subjects at each site, including repeated scans of each subject at a designated home site, is complete. The data acquired will be used to assess the protocols’ inter-site, subject and session reproducibility and variance.

With knowledge of the remaining variance after sequence harmonization it will be possible to identify which sequence modalities would benefit most from work to harmonize results at the analysis stage.

### 4.3 EPI distortion correction

In this work we have carried out EPI distortion correction using FSL's topup routine, with SE-EPI reversed phase-encode blip images providing the estimate of the unwarping to apply. This is done on the basis of previous work (Driver et al., 2018). A simple analysis of the data collected as part of this study replicated the results of that previous work, finding that correction based on field-maps or on topup using SE-EPI measurements provide superior distortion correction to topup correction based on GRE-EPI measurements. This conclusion is made with one caveat. In areas of the brain such as the inferior part of the frontal lobe, which experience large B_0_ offsets, SE-EPI produces very low signal. As such, in these regions Method 3 (field-maps) performs best.

### 4.4 Level of harmonization and limitations

Whilst pulse sequence and reconstruction code were modified for harmonization, it was not currently feasible to harmonize all RF pulses, gradient waveforms and timings precisely. While platform-agnostic pulse sequence development tools are in development (Magland et al., 2016; Ravi et al., 2018) they are not sufficiently mature for all the required applications in this study. Complex sequences, such as EPI multi-band (SMS) were used unmodified on each compatible platform, with just parameters matched. Nor was it possible to harmonize all aspects of reconstruction without reimplementing extensive software libraries and computing hardware. Third party reconstruction software, such as Gadgetron, does offer the possibility of harmonising reconstruction across different types of scanners (Hansen and Sorensen, 2013).

However, harmonization can increase implementation complexity, reducing the ease of initial implementation and reimplementation at subsequent locations. It may also result in compromise towards the “worst-performing” system when specifying protocol performance.

Whilst this work has focused on “key” neuroimaging sequences there are several commonly used sequences which have not been included e.g. diffusion contrast sequences. In future work the UK7T Network will continue to harmonize more complex sequence modalities.

### 4.5 Benefits of harmonization

By publishing these harmonized protocols and data we hope to maximise the possible benefits of sequence harmonization to the 7 T MRI community. Harmonization and subsequent data sharing can help to promote reproducibility of neuroimaging, ease collaboration between sites, and enable the standardisation of analysis tools.

7 T MRI has the ability to greatly enhance our knowledge of brain anatomy, function and metabolism. Disseminating expertise and technology across sites as the number of sites increases is required for 7 T MRI to bring the most benefit to the field of neuroimaging.

## 5 Conclusion

Neuroimaging MRI pulse sequences have been harmonized for the 7 T field strength across three different models of scanners available from two vendors. The protocols have been made publicly available alongside example datasets from a single subject on each of the UK's 7 T scanners. Manual scanner calibration has also been found to be essential for the harmonized use of different scanner models and between sites.

## Supplementary Material

Supporting Information

Appendices

## Figures and Tables

**Table 1:** Scanners and hardware used in the UK7T travelling heads study. The Network comprises five sites, with three different models of scanner, from two different vendors. All scanning used the Nova Medical Inc. (Wilmington MA, USA) single-Tx-channel head coil. The coils were identical except for the coil-scanner interface.

#	Site	Vendor	Scanner Model	Gradient Performance	Installation Date	Software version
1	Wellcome Centre for Integrative Neuroimaging (FMRIB), University of Oxford	Siemens	Magnetom 7 T	70 mT m^−1^	Dec-11	VB17a
				200 mT m^−1^ ms^−1^		
2	Cardiff University Brain Research Imaging Centre, Cardiff University	Siemens	Magnetom 7 T	70 mT m^−1^	Dec-15	VB17a
				200 mT m^−1^ ms^−1^		
3	Sir Peter Mansfield Imaging Centre, University of Nottingham	Philips	Achieva 7 T	40 mT m^−1^	Sep-05	R5.1.7.0
				200 mT m^−1^ ms^−1^		
4	Wolfson Brain Imaging Centre, University of Cambridge	Siemens	Magnetom Terra	80 mT m^−1^	Dec-16	VE11u
				200 mT m^−1^ ms^−1^		
5	Imaging Centre of Excellence, University of Glasgow	Siemens	Magnetom Terra	80 mT m^−1^	Mar-17	VE11u
				200 mT m^−1^ ms^−1^		

**Table 2:** Harmonized structural protocols.

Contrast	Sequence	Resolution (mm^3^)	Field of View (mm^3^)	Coverage	TR (ms)	TE (ms)	BW/px (Hz)	Other	Analysis target		Time (mm:ss)
T1	MPRAGE	0.7 × 0.7 x 0.7	224 × 224 x 224	Whole brain	2200	3.02	240	TI: 1050 m s	Tissue segmentation		06:35
	MP2RAGE	0.7 × 0.7 x 0.7	224 × 224 x 224	Whole brain	3500	2.64	300	TI: 725/2150 m s	Segmentation, R1 mapping		07:51
T2*	GRE	0.7 × 0.7 x 0.7	224 × 224 x 224	Whole brain	31	20	70		SWI, QSM		12:38
	Multi-echo GRE	1.4 × 1.4 x 1.4	269 × 219 x 269	Whole brain	43	4.0–39.0	260	ΔTE: 5.0 m s	QSM, R2* mapping		05:58
T2	TSE	0.4 × 0.4 x 1.0	224 × 224 x 55	Hippocampi	8020	76	155	Turbo factor: 9	Tissue segmentation	2x	04:32
${\text{B}}_{1}^{+}$/Flip-angle	DREAM	4.9 × 4.9 x 4.9	288 × 252 x 288	Whole brain	5000	0.9–1.55	1630	ΔTE: 0.65 m s	${\text{B}}_{1}^{+}$ field calibration		00:05
B_0_ mapping	Dual-echo GRE	4.0 × 4.0 x 4.0	256 × 256 x 256	Whole brain	620	4.08–5.1	730	ΔTE: 0.93 m s	B_0_ field mapping		01:22

**Table 3:** Harmonized functional protocols. MB = multi-band acceleratio n factor; PA = in-plane parallel acceleration (SENSE or GRAPPA).

Contrast	Sequence	Resolution	Field of View	Coverage (# slices)	TR (s)	TE (ms)	BW/px (Hz)	Acceleration	Analysis Target
Resting state fMRI	Multi-band GRE-EPI	1.5 × 1.5 × 1.5 mm^3^	192 × 192 × 144 mm^3^	Whole Brain (96/72†)	1.5	25	1628	MB:3, PA:2‡	Resting state functional connectivity
Task fMRI	Multi-band GRE-EPI	1.5 × 1.5 × 1.5 mm^3^	192 × 192 × 84 mm^3^	Motor-visual (56)	2	25	1954	MB:2, PA:2	Task fMRI (motor & visual task)
	GRE-EPI	1.5 × 1.5 × 1.5 mm^3^	192 × 192 × 51 mm^3^	Primary motor (34)	2	25	1954	PA:2	Task fMRI (motortopy)
Distortion correction	SE-EPI	Same as fMRI	Same as fMRI	Same as fMRI	3	45	Same as fMRI		Distortion correction
${\text{B}}_{1}^{+}$/Flip-angle	DREAM	4.9 × 4.9 × 4.9 mm^3^	288 × 252 × 288 mm^3^	Whole brain	5	0.9/1.55	1630	PA:2	${\text{B}}_{1}^{+}$ field calibration

†,‡ Due to gradient heating limitations, Site 3 (Table 1) was unable to acquire as many slices in the resting state protocol whilst maintaining the same total scan time (10.5 min), in the same scanner in-plane acceleration was reduced to 1.5 from 2.
