# Supplementary material for: Multi-site harmonization of 7 tesla MRI neuroimaging protocols
Source: Neuroimage. Author manuscript; Available in PMC 2020 May 11. (PMC7212005; doi:10.1016/j.neuroimage.2019.116335)
Supplement: Supporting Information [file EMS86317-supplement-Supporting_Information.zip › 1-s2.0-S1053811919309267-mmc1.docx]

Supporting information


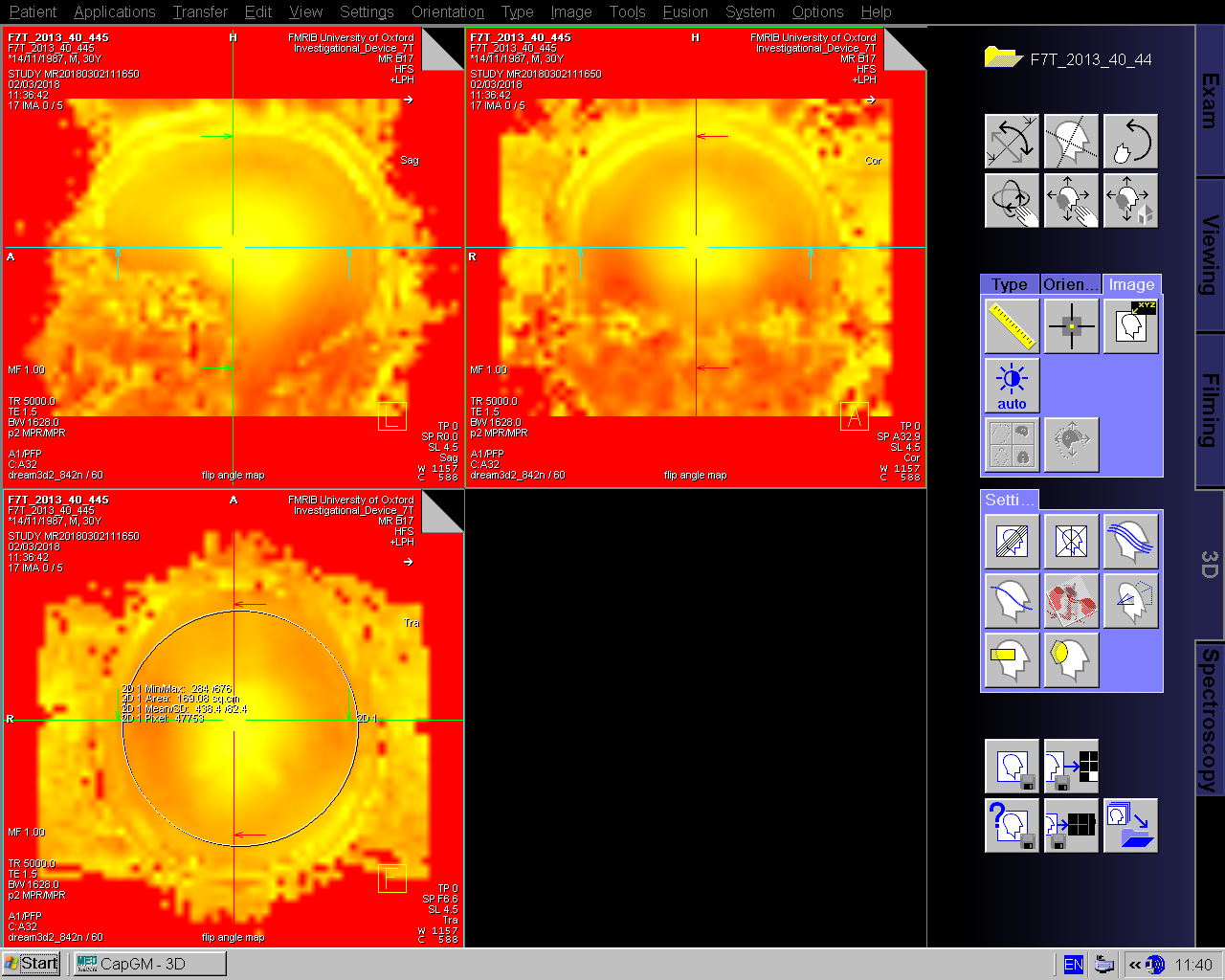

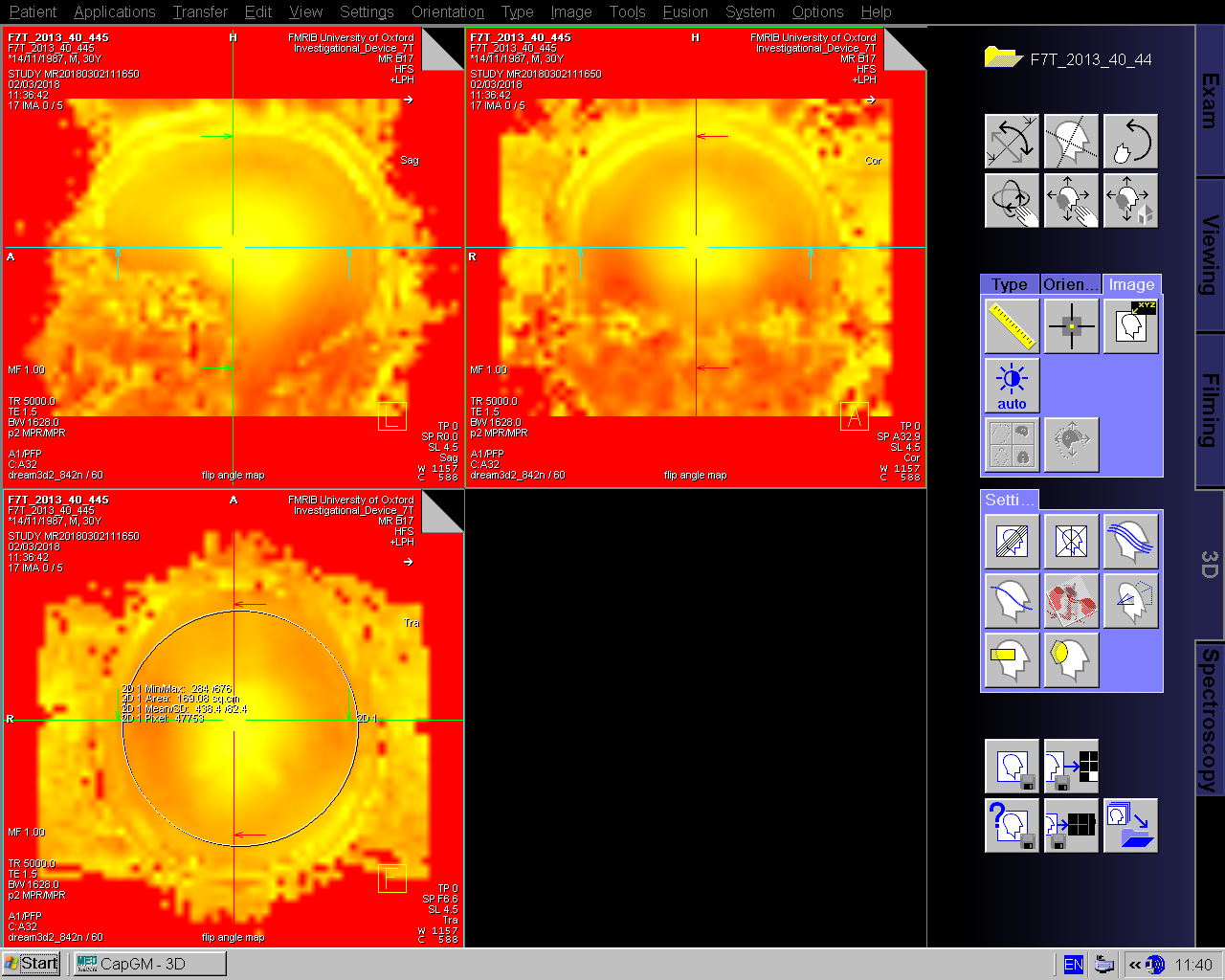


**Supporting Figure 1:** Sagital (L) and transverse (R) views of the DREAM flip-angle map in the 3D viewer on a Siemens scanner. The circular ROI is shown drawn in the right-hand panel.

**Supporting Figure 2:** (Top) Difference between whole brain and slice mean B_1_^+^ for all subjects (different colours). Slices 17-20 all show low differences. (Bottom) 2-Norm of the top panel across all subjects.
