# Supplementary material for: Multi-site harmonization of 7 tesla MRI neuroimaging protocols
Source: Neuroimage. Author manuscript; Available in PMC 2020 May 11. (PMC7212005; doi:10.1016/j.neuroimage.2019.116335)
Supplement: Appendices [file EMS86317-supplement-Appendices.pdf]

Appendix A. Task fMRI protocols

1. Motor-visual task: 4 min of 20s ON/20s OFF, iso-luminant 2 Hz reversing radial checkerboard. Visual angles were 24°/19°/20°/14°/30° for Sites 1–5. The motor task was a unilateral fingers-to-thumb motion, visually paced at 2 Hz and alternating between left- and right-hand movement across consecutive ON periods.
2. Motortopy task: 2 × 4.5 min of 1 Hz sequential single-digit button pressing. Each of the four digits was visually cued at 1 Hz for 8 s for 8 cycles (32 s per cycle, 256 s total). In the second acquisition the order of the digits was reversed. This design matched that described in Kolasinski et al. (2016).

Appendix B. Implementation of Roemer Coil Combination

The uniform sensitivity Roemer coil combination was implemented as in Roemer et al. (1990) & Pruessmann et al. (1999). The process is divided into two stages. In the first stage, coil sensitivity and noise correlation information are acquired using additional short reference scans prior to the acquisitions which will use the Roemer coil combination. In the second stage, run as part of the online reconstruction of images, the coil sensitivities and noise correlation information previously calculated is used to combine the individual coil images. The steps within each stage are given in more detail below.

Calculation of coil sensitivities

1. Two 3D gradient recalled echo (GRE) scans with the following near-identical parameters are acquired. FOV: 360 × 360 × 360 mm^3, resolution: 5.6 × 5.6 × 5.0 mm^3, BW: 1000 Hz/px, TA: 0:29, flip-angle: 5°, water only excitation. The first acquisition uses the 32-channel array to receive the MR signal and the second uses the volume birdcage coil. In addition to the main image acquisition the first scan also contains 10 s of noise measurements before the acquisition. The protocols for the calibration scans are available as part of the deposit (Clarke, 2018).
2. A noise correlation matrix for the 32-channel array is calculated from the 10 s of noise data per channel (dwell time = 10 μs, 1 million points). The noise correlation matrix is stored in memory for use later.
3. The coil sensitivity data is calculated, masked, fitted using polynomials and stored. After the vendor's own image reconstruction steps (3D Fourier transform etc.) the following additional steps are performed on each 2D partition separately:
   a. The image from each individual receive array coil are divided by the single image received on the volume birdcage coil. This produces 32 individual raw coil sensitivity images.
   b. A sum-of-squares reconstruction of the array coil images is used to create a tissue mask by thresholding. The threshold is set to 10 times the noise value in the first partition in the inferior part of the image rising to 30 times in the superior part of the image. This mask is applied to the raw coil sensitivity images. A region growing algorithm is applied to the image to extend the coil sensitivity maps beyond the area of the sample. This is achieved using a 3 × 3 uniformly weighted blurring kernel to replace missing values resulting from thresholding within and around the edge of the tissue region.
   c. The masked coil sensitivity images are then fitted using polynomials (up to 4th order) by solving equation (26) in Pruessmann et al. (1999). The problem is weighted according to equation (29) in Pruessmann et al. (1999), using an $\Omega = 15$. The minimisation is implemented using QR factorisation in the LAPACK "gels" function (Anderson, 1999).
   d. The fitted coil sensitivity images are then stored for later use.

Coil combination

The following steps are completed at the end of the reconstruction chain for a 3D image using the Roemer combination. The first six steps are completed partition-wise on 2D images and assembled as a full 3D image in the final stage.

1. The fitted sensitivity maps are loaded from memory.
2. The loaded sensitivity maps undergo trilinear interpolation to the imaging resolution and orientation.
3. The coil noise covariance matrix is loaded from memory, scaled to the imaging bandwidth, inverted and conjugated.
4. The interpolated sensitivity images and raw imaging data are passed to the Roemer combination calculation routine. In this function the uniform sensitivity Roemer combination is carried out in a vectorised way on all pixels simultaneously for computational speed reasons (Roemer et al., 1990).
5. The combined complex image is split into magnitude and phase images.
6. Scaling using an empirically tuned constant value is applied to ensure the magnitude image range is within that of 13 (12 + 1) bit DICOM images (±4096).
7. The process is repeated for all partitions in the 3D image, the image is assembled and exported by the scanner's normal reconstruction pipeline.

Appendix C. B_1^+ calibration using DREAM flip-angle mapping

The following instructions were included in scanning procedures for Siemens scanners. The Philips scanner followed a procedure adapted for the different user interface (different display units, 2D viewer and greyscale display). The 3D DREAM sequence was run in the following position and orientation:

Position: Whole head coverage. Orientation: Axial

Angle: None.

After running the flip-angle mapping, the generated flip-angle map was loaded into the online 3D viewer. This shows a colour-map of the flip-angle measured in the whole head (units = degrees x 10).

Position the 3D view so that you can see a wholly transverse view at the point where the head is largest in the AP direction (Supporting Fig. 1, left). Use the circle ROI tool to draw an ROI encompassing as much brain as possible in the transverse view, without encroaching on non-brain tissue (Supporting Fig. 1, right).

Read the mean value. On Siemens scanners make the following calculation:

$$V_{\mathrm{Ref}} = 250^{*}\ \frac{\alpha FA_{\mathrm{target}}}{Mean\ FA_{\mathrm{ROI}}}$$

V_{Ref} is the “reference voltage”. This value is applied as the transmit gain calibration value. On the Philips scanner an equivalent calculation is made where V_{Ref} is replaced by the drive scale. FA_{target} is the target flip-angle of the preparation pulses of the DREAM sequence. α is a scaling factor which tunes the calibration to a fraction of the target e.g. $\alpha = 0.8$ will result in the mean flip-angle achieved being 80% of the target. This is included to ensure that the SAR limit is not breached in subjects with low B_1^+ per unit drive voltage.

In the example Supporting Fig. 1 the target flip-angle is 60° and $\alpha = 0.8$.

$$V_{\mathrm{Ref}} = 250^{*}\ \frac{480}{438} = 274\ V$$

On Siemens Magnetom 7 T scanners a value between 260 and 300 V was typically realised.

Justification

This method was validated from a three-site, three-subject pilot study. In this study it was observed that for all subjects on all scanners the mean B_1^+ in mid-axial slices was <10% of the whole brain mean B_1^+. Therefore, transmit calibration could be accurately made using the simple online ROI drawing tools.

Appendix D. Supplementary data

Supplementary data to this article can be found online at https://doi.org/10.1016/j.neuroimage.2019.116335.
